# Role of Disulfide Bonds in Activity and Stability of Tigerinin-1R

**DOI:** 10.3390/ijms19020288

**Published:** 2018-01-23

**Authors:** Xiaolong Chen, Cuihua Hu, Yibing Huang, Yuxin Chen

**Affiliations:** 1 Key Laboratory for Molecular Enzymology and Engineering of the Ministry of Education, Jilin University, Changchun 130012, China; xlchen2017@sinano.ac.cn (X.C.); hucvihua@163.com (C.H.); huangyibing@jlu.edu.cn (Y.H.); 2 College of Life Sciences, Jilin University, Changchun 130012, China

**Keywords:** tigerinin-1R, INS-1, Ca^2+^ influx, disulfide bonds, oxidative stress

## Abstract

Tigerinin-1R (Arg–Val–Cys–Ser–Ala–Ile–Pro–Leu–Pro–Ile–Cys–His–NH_2_), a cationic 12-mer peptide containing a disulfide bond extracted from frog skin secretions, lacks antibacterial activity, but has the ability to stimulate insulin release both in vitro and in vivo. To study the structure–function relationships of tigerinin-1R, we designed and synthesized five analogs, including tigerinin-cyclic, tigerinin-1R-L4, tigerinin-linear, [C3K]tigerinin-1R, and [C11K]tigerinin-1R. Tigerinin-1R promoted insulin secretion in a concentration-dependent manner in INS-1 cells without obvious cytotoxicity. At a concentration of 10^−5^ M, [C11K]tigerinin-1R exhibited the highest stimulation ability, suggesting that the positive charge at the C-terminus may contribute to the in vitro insulin-releasing activity of tigerinin-1R. Tigerinin-1R peptides stimulated insulin release in INS-1 cells through a universal mechanism that involves mobilization of intracellular calcium without disrupting the cell membrane. In vivo experiments showed that both tigerinin-1R and [C11K]tigerinin-1R improved glucose tolerance in overnight-fasted mice. Due to its structural stability, tigerinin-1R showed superior hypoglycemic activity to [C11K]tigerinin-1R, which suggested a critical role of the disulfide bonds. In addition, we also identified a protective effect of tigerinin-1R peptides in apoptosis induced by oxidative stress. These results further confirm the potential for the development of tigerinin-1R as an anti-diabetic therapeutic agent in clinical practice.

## 1. Introduction

Diabetes mellitus (DM) is a group of metabolic diseases characterized by chronic hyperglycemia [1,2]. As the morbidity of diabetes increases, it has become one of major threats to human health [3]. In all types of diabetes, type 2 diabetes is the main type, and accounts for more than 90% of diabetic patients [4,5]. One treatment approach is the use of agents based on physiological incretins, as patients with type 2 diabetes show a relative lack of insulin [6]. Another is the use of agents to protect pancreatic β-cells from damage caused by oxidative stress, as the expression of various enzymes of the anti-oxidation defense system is low in type 2 diabetes patients [7,8,9].

A peptide is a relatively small “protein”, composed of less than 100 amino acids, with low cost, high biological activity and specificity, low immunity, and fast absorption [10,11]. Recently, several insulin-releasing peptides, including glucagon-like peptide-1 (GLP-1), glucose-dependent insulinotropic peptide (GIP), exenatide, and liraglutide (two analogs of GLP-1), have been extensively studied. Among these, exenatide and liraglutide are two peptide drugs that have been commercialized for clinical treatment, and have been shown to benefit many patients with diabetes [12,13,14,15]. In addition to these peptides, skin secretions of anurans as a natural peptide library have recently received increasing attention [16,17]. Several peptides, such as temporin-Vb [18], esculentins-1, esculentins-1B, brevinins-1E, and brevinins-2EC [19], which were isolated as antimicrobial agents, were shown to promote the secretion of insulin in vitro or in vivo. However, peptides such as tigerinin-1R [20] and alyteserin-2a [21], which are partially deficient in antimicrobial activity, are able to promote the secretion of insulin, showing a potential for use in the treatment to type 2 diabetes. It seems that there is no relation between their insulin-releasing activity and antimicrobial activity.

Tigerinin-1R (Arg–Val–Cys–Ser–Ala–Ile–Pro–Leu–Pro–Ile–Cys–His–NH_2_) is a cationic peptide containing a disulfide bond that is isolated from the skin secretions of *Hoplobatrachus ugulosus*. It lacks antibacterial activity, but is considered to be a potent, non-toxic insulin-releasing peptide [20,22]. In vitro studies have shown that it stimulates the release of insulin, even at concentrations as low as 0.1 nM, and exhibits no hemolytic activity against human erythrocytes at concentrations up to 500 nM [22]. In vivo studies have shown that administration of tigerinin-1R or some of its analogs to high-fat fed mice significantly enhanced insulin release and improved glucose tolerance [20,22,23,24]. Ojo et al. suggests that a C-terminally amidated amino acid is necessary for the potency of the peptide. The mechanism underlying the promotion of insulin secretion in BRIN-BD11 cells involves depolarization of the membrane and influx of calcium [22]. As tigerinin-1R exhibits great potential as a therapeutic agent for diabetes, in this study, we carried out a series of modifications based on the structure of tigerinin-1R to explore its structure–activity relationships. For the in vitro activity experiments, the islet cell line INS-1, which is more readily available, was used to demonstrate the structure–activity relationships and mechanism of tigerinin-1R. The protective effect of the tigerinin-1R peptides on palmitic acid-induced high-fat injury was also studied.

## 2. Results

### 2.1. Peptide Design and Structure

In the present study, peptide tigerinin-1R was used as a framework to systematically alter the sequence. Eventually, five tigerinin-1R analogs were obtained and termed tigerinin-cyclic, tigerinin-1R-L4, tigerinin-linear, [C3K]tigerinin-1R, and [C11K]tigerinin-1R. The sequences and the relative hydrophobicity of the peptides are shown in Table 1. The non-polar residues Ala, Ile, and Ile at positions 5, 6, and 10, respectively, were replaced by more hydrophobic leucine residues in tigerinin-1R-L4. The amino acids Arg, Val, His at both ends of tigerinin-1R were removed, and only the cyclic moiety was retained in the structure referred to as tigerinin-cyclic. In tigerinin-linear, both cysteine residues at positions 3 and 11 were replaced by alanine, while in [C3K]tigerinin-1R and [C11K]tigerinin-1R, the cysteine residues at position 3 or 11, respectively, were replaced by a positively charged lysine.

**Figure 1:**
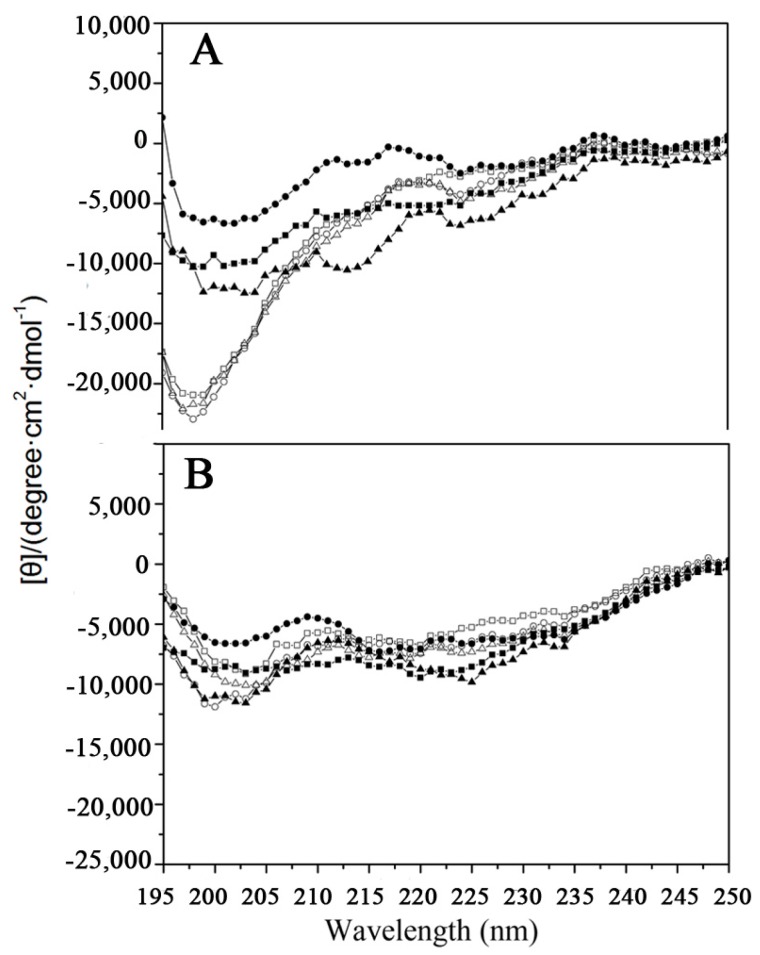
Circular dichroism spectra of the peptides. Panels A and B show the CD spectra of peptides in KP buffer and in the presence of 50% (*v*/*v*) TFE, respectively. Symbols used are as follows: solid square ■ for tigerinin-1R; solid circle ● for tigerinin-1R-L4; solid triangle ▲ for tigerinin-cyclic; open square □ for tigerinin-linear; open circle ○ for [C3K]tigerinin-1R; and open triangle △ for [C11K]tigerinin-1R.

As shown in Figure 1, the secondary structures of the peptides were measured by circular dichroism spectroscopy under benign conditions (potassium phosphate (KP) buffer, mimicking the hydrophilic environment) (Figure 1A) and in KP buffer with 50% trifluoroethanol (TFE) to mimic the hydrophobic environment (Figure 1B). All the peptides exhibited a random coil structure under benign conditions. By contrast, the peptide structures in the presence of 50% TFE was quite different compared to that in aqueous medium (Figure 1). A previous study showed that tigerinin-1R adopted a conformation comprising a mixture of β-sheets (40%), random coils (48%), and type I reverse β-turns (12%) in 50% aqueous TFE [20]. The results of the circular dichroism (CD) experiments are consistent with the previous report. Although tigerinin-linear, [C3K]tigerinin-1R, and [C11K]tigerinin-1R adopt linear forms, they exhibited a structure similar to tigerinin-1R, tigerinin-cyclic, and tigerinin-1R-L4 under hydrophobic conditions, indicating that the structure may be of great importance to their activities.

### 2.2. Cellular Toxicity of Peptides

**Figure 2:**
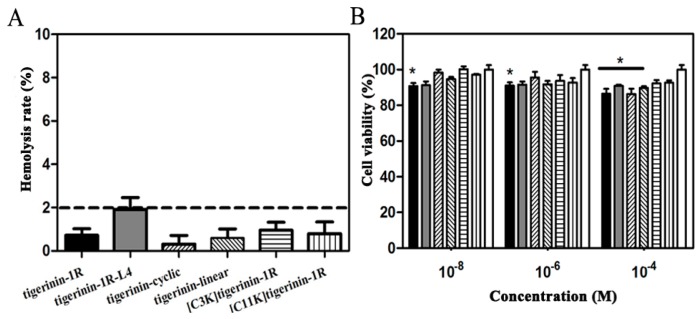
Cellular toxicity of the peptides. (**A**) The calculated hemolysis rate of peptides of 250 μM after incubation with 70 μL human erythrocytes for 2 h. Hemolysis was denoted as the values greater than 2% (the dotted line). (**B**) The viability of INS-1 cells following incubation with peptides of different concentrations for 24 h. Columns used are as follows for both graphs: black solid column for tigerinin-1R; gray solid column for tigerinin-1R-L4; left slash filled column for tigerinin-cyclic; right slash filled column for tigerinin-linear; horizontal line filled column for [C3K]tigerinin-1R;vertical line filled column for [C11K]tigerinin-1R; open column for negative control (* *p* < 0.05 compared with negative control; *n* = 3).

The hemolytic activity of the peptides against human erythrocytes was determined as a measure of peptide toxicity against normal cells. None of the peptides exhibited hemolysis against human red blood cells at a concentration of 250 μM after 2 h of incubation at 37 °C, as the calculated hemolysis rate was less than 2% (Figure 2A). The rat insulinoma INS-1 cell line, which is widely used in insulin secretion studies, has been used as a model for the in vitro study of biological activities of tigerinin analogs [25,26,27]. As shown in Figure 2B, 3-(4,5-dimethylthiazol-2-yl)-2,5-diphenyltetrazolium bromide (MTT) tests confirmed that no tigerinin peptide exhibited obvious cytotoxicity against INS-1 cells at concentrations of 10^−8^, 10^−6^, and 10^−4^ M after incubation for 24 h at 37 °C.

### 2.3. Insulin-Release Activity of Peptides In Vitro

**Figure 3:**
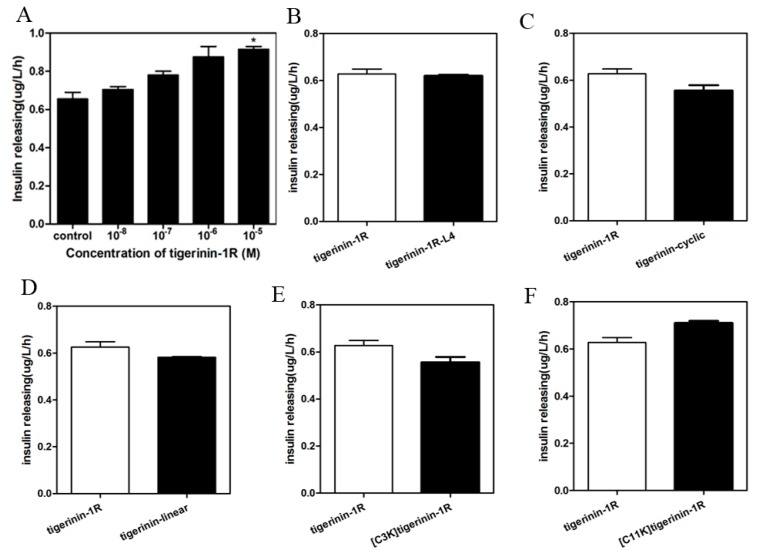
Effect of peptides on the release of insulin from INS-1 cells in the presence of 2.8 mM glucose. (**A**) INS-1 cells were treated with different concentrations of tigerinin-1R in the presence of 2.8 mM glucose (* *p* < 0.05 compared with glucose alone; *n* = 3). (**B**–**F**) INS-1 cells were treated with peptide analogs at a concentration of 10^−5^ M in the presence of 2.8 mM glucose. The assays were repeated in triplicate.

As shown in Figure 3A, the parent peptide tigerinin-1R exhibited insulin-releasing activity in rat insulinoma INS-1 cells after incubation for 1 h in 1 mL KRB buffer (115 mM NaCl, 4.7 mM KCl, 1.28 mM CaCl_2_, 1.2 mM KH_2_PO_4_, 1.2 mM MgSO_4_, 10 mM NaHCO_3_, and 1 g/L BSA, pH 7.4) at 37 °C. Tigerinin-1R increased insulin secretion of INS-1 cells at different concentrations compared to 2.8 mM glucose alone. It showed a concentration-dependent manner between concentrations of 10^−8^ to 10^−5^ M. As tigerinin-1R exhibited no toxicity and the highest insulin-promoting activity at a concentration of 10^−5^ M in the gradient series, this concentration was chosen to evaluate the insulin-release activity of the analogs. As shown in Figure 3B, the level of insulin release in the tigerinin-1R-L4 group was similar to that of the parent peptide, suggesting that increased hydrophobicity does not necessarily have an effect on the insulin-releasing activity. Compared to tigerinin-1R, tigerinin-cyclic retains only the cyclic moiety, without the hydrophilic positive amino acid “tail” at both ends. The decrease in insulin-releasing ability of tigerinin-cyclic indicates that the positively charged amino acids may contribute to the insulin-releasing ability of tigerinin-1R (Figure 3C). Tigerinin-linear with two Ala substitutions instead of two Cys residues, and [C3K]tigerinin-1R with a single Lys replacing Cys at position 3, showed lower insulin-releasing activity than tigerinin-1R (Figure 3D,E). By contrast, [C11K]tigerinin-1R, with a Lys residue replacing Cys at position 11, showed increased insulin-releasing ability than tigerinin-1R (Figure 3F). It seems that the disulfide bonds play an important, but not irreplaceable role in the insulin-releasing activity of tigerinin-1R in vitro. Increasing the positive charge of tigerinin-1R at the C-terminus could promote the insulin-releasing capacity.

### 2.4. Mechanism of Stimulation of Insulin Release

**Figure 4:**
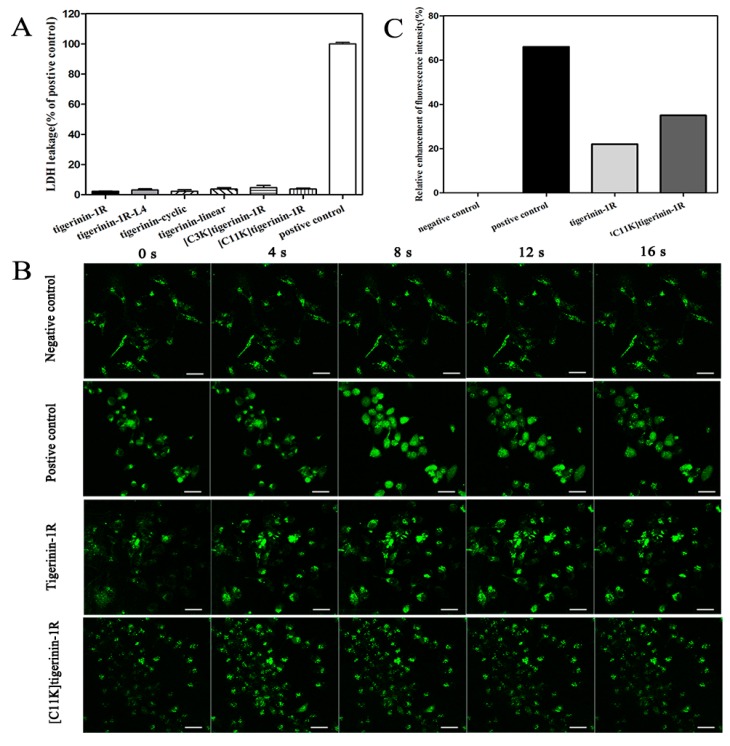
Mechanism of stimulation of insulin release. (**A**) Lactate dehydrogenase leakage after incubation with 10^−5^ M peptide in the presence of 2.8 mM glucose for 1 h. 1% Triton X-100 served as the positive control. The assays were repeated in triplicates. (**B**) INS-1 cells were loaded with 5 μM Fluo-3AM. [Ca^2+^]*_i_* indicated by the fluorescence intensity was measured. The fluorescence was excited at 480 nm at 4 s intervals and detected at 515 nm. Glucose (2.8 mM) alone (negative control), 30 mM KCl (positive control), or peptides of 10^−5^ M supplemented with 2.8 mM glucose were added, respectively. Scale bar = 100 μm and refers to all panels. (**C**) The relative enhancement of fluorescence intensity was calculated as (the maximum arithmetic mean intensity value − the initial arithmetic mean intensity value)/the initial arithmetic mean intensity value × 100%. The arithmetic mean intensity values were quantitated using ZEN 2012 image software (CarlZeiss, Oberkochen, Germany).

As shown in Figure 4A, the increase in lactic acid dehydrogenase (LDH) was not detected in the cell supernatant after 1 h incubation with peptides at a concentration of 10^−5^ M, indicating that the increase in insulin release was not caused by the destruction of the cytoplasmic membrane. With a glucose concentration of 2.8 mM, both tigerinin-1R and [C11K]tigerinin-1R produced an increase in the concentration of intracellular Ca^2+^ in INS-1 cells (Figure 4B,C). The magnitude of the effect was less than that produced by 30 mM KCl (positive control), but was significantly greater than that in the presence of glucose alone (negative control). Both peptides caused sharp initial increases in the concentration of intracellular calcium, which can be observed between 4 and 12 s in the fluorescence study (Figure 4B). Although Ca^2+^ plays an important role in the pathway of insulin exocytosis [6], this data indicates that tigerinin-1R and [C11K]tigerinin-1R stimulated insulin secretion via Ca^2+^ influx in INS-1 cells.

### 2.5. Effect of Tigerinin-1R and [C11K]Tigerinin-1R on Glucose Tolerance In Vivo

As shown by the results of the in vitro experiments, [C11K]tigerinin-1R exhibited the strongest insulin-releasing efficiency, followed by tigerinin-1R and tigerinin-1R-L4. By contrast, the stimulation efficiency of the other peptides was lower than that of tigerinin-1R. Thus, [C11K]tigerinin-1R and tigerinin-1R, the two most potent insulin-releasing agents as representatives of linear and disulfide-cyclized peptides respectively, were selected to study the in vivo activity.

**Figure 5:**
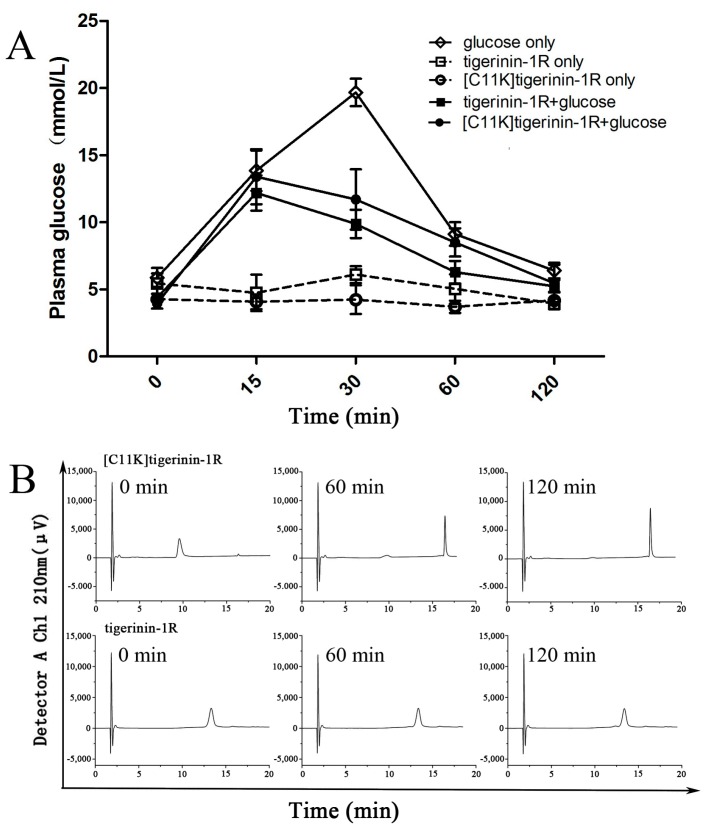
In vivo study and stability of peptides in 10% fetal bovine serum. (**A**) Effect on plasma glucose concentrations of tigerinin-1R and [C11K]tigerinin-1R (75 nmol/kg body weight) in lean mice. Glucose (18 mmol/kg body weight) was administered with or without the peptides by intraperitoneal injection. Values are mean ± SEM (*n* = 6). (**B**) Stability of tigerinin-1R and [C11K]tigerinin-1R in 10% fetal bovine serum. The remaining intact peptides were detected by reversed-phase high performance liquid chromatography (RP-HPLC) after incubation with 10% fetal bovine serum for different times.

As shown in Figure 5A, there was little change in plasma glucose concentrations in mice treated with tigerinin-1R or [C11K]tigerinin-1R alone after overnight fasting, without intraperitoneal injection of high glucose. Meanwhile, plasma glucose concentrations of the mice injected with glucose alone increased at 15 and 30 min, but decreased sharply at 60 min. When the high concentration of glucose was received in combination with tigerinin-1R or [C11K]tigerinin-1R, the two groups showed the same trend, that is, the value of plasma glucose concentrations increased at 15 min, and decreased at 30, 60, and 120 min. Interestingly, tigerinin-1R showed higher hypoglycemic ability than [C11K]tigerinin-1R, as the blood glucose concentration values of the tigerinin-1R group at 30 and 60 min were lower than those of [C11K]tigerinin-1R. This appeared to differ from the results of the in vitro insulin release test, which may be due to the difference in peptide stability in vivo. Subsequent peptide degradation assays demonstrated this hypothesis. The stability of tigerinin-1R and [C11K]tigerinin-1R was determined in 10% fetal bovine serum (Figure 5B). The stability of [C11K]tigerinin-1R was clearly much worse than that of tigerinin-1R. The peak area of tigerinin-1R changed very little within 120 min during RP-HPLC; by contrast, the peak area of [C11K]tigerinin-1R decreased dramatically after 60 min and had almost disappeared at 120 min. This result was consistent with the results of the in vivo experiments, indicating that the presence of disulfide bond was of great importance to the stability of tigerinin-1R.

### 2.6. Protective Effects against Apoptosis Induced by Palmitic Acid

**Figure 6:**
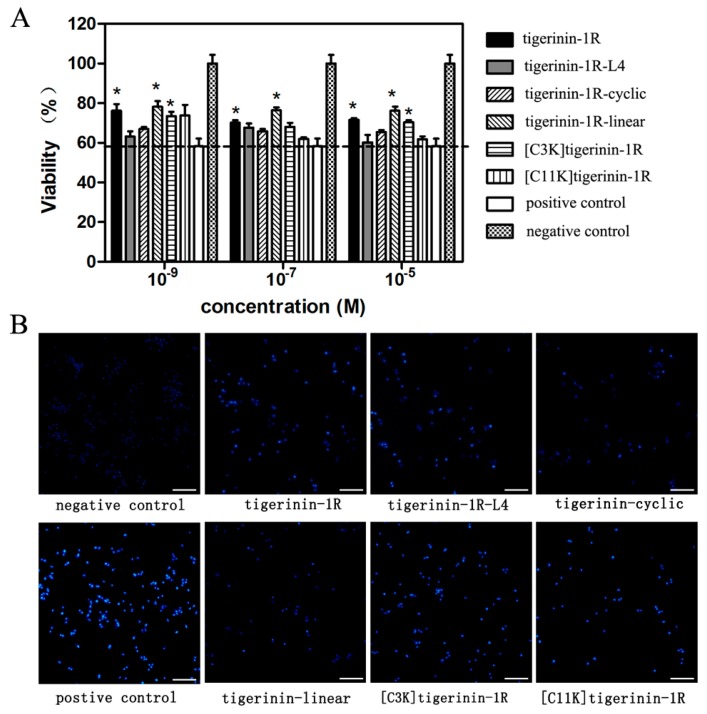
Protective effects of peptides against apoptosis induced by PA. (**A**) INS-1 cells were treated with different concentrations of peptides in the presence/absence of 0.5 mM PA for 24 h. The viability was tested by MTT assays. The value at the dotted line is equal to the group of positive control. (* *p* < 0.05 compared with positive control; *n* = 3) (**B**) INS-1 cells were treated with 10^−5^ M peptides in the presence/absence of 0.5 mM PA for 24 h. Nuclei were stained with Hoechst 33258 (in blue color). Scale bar = 100 μm and refers to all panels.

It has been reported that palmitic acid (PA) may induce the apoptosis of INS-1 cells [7,28]. Therefore, a PA-induced cell damage model was used to examine whether tigerinin-1R peptides possess the ability to protect cells from this state. As shown in Figure 6A, the viability value of INS-1 cells incubated with 0.5 mM PA for 24 h (the positive control) was 58.24%, showing the successful induction of apoptosis by PA. The viability values of cells cultured in the presence of peptide analogs at concentrations of 10^−9^, 10^−7^, and 10^−5^ M, respectively, increased to different degrees compared to that of the positive control. The results showed that tigerinin-1R peptide analogs exhibited protective effects against apoptosis induced by PA under appropriate concentrations. Hoechst 33258 staining was then performed to further verify the protective effects. As in Hoechst 33258 staining, the apoptotic nuclei observed by fluorescence microscopy showed highly concentrated chromatin compared to normal cells. Cells treated with 0.5 mM PA (positive control) showed brighter nuclei, while the negative control showed the darkest nuclei. Cells treated with 0.5 mM PA in the presence of tigerinin-1R peptides of 10^−5^ M showed darker nuclei than positive control, but brighter than the negative control (Figure 6B). Together, these data suggested a protective effect of tigerinin-1R peptides against PA-induced apoptosis.

## 3. Discussion

The increasing morbidity of type 2 diabetes mellitus has necessitated a search for new types of therapeutic agents with the ability to stimulate the release of insulin. Among them, peptide drugs have received much attention, due to their unique advantages. Two analogs of GLP-1, exenatide and liraglutide, have been successfully applied to clinical practice to the benefit of thousands of diabetic patients [15,29,30]. Moreover, skin secretions of anurans as a natural peptide library have yielded a variety of peptide candidates, among which several agents have been shown to have the ability to promote insulin release [16,17,18,19,20,21].

Although the relationship between the structure and activity of insulinotropic peptides is not yet fully understood, a previous study showed that the C-terminal disulfide loop of gaegurin-6 was crucial for its biological activities [31]. However, for brevinin-2, the cyclic heptapeptide domain at the C-terminus was not necessary for its biological activities [32]. Increasing the hydrophobicity of alyteserin-2a had a deleterious effect on its insulinotropic activity [21]. In contrast, when increasing the hydrophobicity of tigerinin-1R by substituting amino acids with more hydrophobic tryptophan residues, all Trp-containing analogs were more potent than the native peptide [20]. All of the above results were obtained using BRIN-BD11 cells. In contrast, in this study, a more easily available insulin-secreting model INS-1 cell was used. It is interesting to see that tigerinin-1R-L4, which shows increased hydrophobicity compared to the parent peptide, did not show any differences in insulinotropic activity, which indicates that there is no correlation between hydrophobicity and insulin-releasing activity. The insulinotropic activity of tigerinin-cyclic was lower than that of tigerinin-1R, indicating that positively charged amino acid “tail” outside the disulfide ring structure contributes to the activity of tigerinin-1R. [C3K]tigerinin-1R has a similar structure to [C11K]tigerinin-1R, that is, one cysteine residue participating in the formation of disulfide bond was replaced by a positively charged lysine. However, the effects on insulin release of these two analogs were quite different. The insulin-releasing activity of [C3K]tigerinin-1R was lower than tigerinin-1R, while that of [C11K]tigerinin-1R was higher. This result indicates that the positive charge at the C-terminus rather than N-terminus contributed to the insulin-releasing activity of tigerinin-1R.

Previous studies have shown that tigerinin-1R and analogs stimulated insulin release mainly through a mechanism involving the mobilization of intracellular calcium [20,22]. Gaegurin-6 is an example with such a mechanism. It increased the concentration of intracellular calcium under normal conditions, but failed to do so in the presence of ethylenediaminetetraacetic acid (EDTA) [31]. In contrast, the stimulatory effects of esculentin-1 and esculentin-1B were mediated by a combination of pathways involving both protein kinase A (PKA) and protein kinase C (PKC), which were not affected by 50 µM verapamil [19]. Interestingly, some peptides can even promote the secretion of GLP-1, which promotes the secretion of insulin [33]. Lactate dehydrogenase (LDH) is an intracellular enzyme that does not leak out when the cell membrane is intact [34]. In our study, an increase in LDH was not detected in the cell supernatant after one h of incubation with peptides at a concentration of 10^−5^ M, indicating that the increase in insulin release was not caused by the destruction of the cell membrane. Fluo-3 AM is a type of fluorescent probe used for detecting the concentration of intracellular calcium [35]. It can penetrate the cell membrane and transform into Fluo-3, which combines with calcium ions, producing strong fluorescence that instantly reflects changes in the concentration of calcium ions. Our results show that both tigerinin-1R and [C11K]tigerinin-1R can cause an influx of calcium ions, meaning that the mechanism for insulin-promotion activity in INS-1 cells is partially related to the mobilization of intracellular calcium. In addition, compared to tigerinin-1R, [C11K]tigerinin-1R caused a stronger calcium influx, which is consistent with the results of insulin release in vitro, as [C11K]tigerinin-1R showed a greater insulin-releasing ability compared with tigerinin-1R.

Glucose tolerance refers to the ability to regulate blood glucose levels, reflecting the body’s ability to release insulin [23]. The in vivo study in Kunming male mice indicated that after intraperitoneal injections of tigerinin-1R and [C11K]tigeinin-1R at 75 nmol/kg body weight, glucose tolerance was significantly improved without serious adverse effects. However, due to the stronger stability of its structure, the glucose tolerance activity of tigerinin-1R was better than that of [C11K]tigerinin-1R in vivo. It has been reported that the cyclic structure is more stable than the linear structure [36,37]. Thus, the presence of disulfide bonds is important for the in vivo activity of tigerinin-1R, as a better stability implies a longer duration of action.

As reported previously, pancreatic β-cells are extraordinarily sensitive to oxidative stress due to much lower levels of superoxide dismutase, catalase, and glutathione peroxidase gene expression compared with other tissues [9]. However, chronic elevated free fatty acids (FFAs) activate stress sensitive intracellular signaling pathways, and result in oxidative stress via various mechanisms, such as β-cell mitochondrial dysfunction, and the production of reactive oxygen species and reactive nitrogen species [7]. Therefore, protection of β-cells from oxidative stress has been regarded as one strategy for the treatment of type 2 diabetes. To study the effect of tigerinin-1R analogs on the protection of pancreatic β-cells, a 0.5 mM FFA solution was used to develop a model of hyperlipidemia [8,38]. The cell viability values of peptide-treated groups were greater compared to the positive control, suggesting a protective effect of tigerinin-1R peptides on FFA-induced oxidative stress in vitro. Therefore, in addition to promoting the release of insulin, the improvement of the oxidative stress of pancreatic β-cells in type 2 diabetes by long-term treatment with tigerinin-1R peptides could be expected.

## 4. Materials and Methods

### 4.1. Peptide Synthesis and Purification

Synthesis of the peptides was carried out by solid-phase peptide synthesis using 9-fluorenyl-methoxycarbonyl (Fmoc) chemistry and methylbenzhydrylamine (MBHA) Rink amide resin (0.629 mmol/g) [39]. Disulfide-based cyclic peptides were oxidized by air after the linear-chain synthesis completed. Purification was then carried out by reversed phase high-performance liquid chromatography (RP-HPLC) as described previously [40]. The purity of peptides was verified by analytical RP-HPLC and further characterized by mass spectrometry and amino acid analysis.

### 4.2. Circular Dichroism

Circular dichroism (CD) measurements were performed using a 0.02 cm path length quartz cuvette on a J-810 CD spectropolarimeter (Jasco, Tokyo, Japan) at room temperature. The peptides were dissolved in (a) KP buffer (50 mM KH_2_PO_4_, K_2_HPO_4_, 100 mMKCl, pH 7.4) and (b) 50% trifluoroacetic acid (TFE) in KP buffer at a concentration of 75 μM. Data were collected from 190 to 250 nm. The mean residue molar ellipticities were calculated as described previously [34].

### 4.3. Measurement of Hemolytic Activity

Peptides were serially diluted in PBS (137 mM NaCl, 2.7 mM KCl, 10 mM Na_2_HPO_4_, 2 mM KH_2_PO_4_, pH 7.4), and a volume of 70 μL sample solution/well was transferred to round-bottomed 96-well plates. After incubation with 70 μL human erythrocytes for 2 h, aliquots from the hemolysis assays were withdrawn, and unlysed erythrocytes were precipitated by centrifugation (3000 rpm, 4 °C, 5 min). Hemoglobin release was determined spectrophotometrically at 578 nm. Erythrocytes in PBS were used as a negative hemolysis control (0%) and in distilled water as a positive hemolysis control (100%). The assay was repeated in triplicate.

### 4.4. Cell Culture and Cytoxicity Assays

INS-1 rat insulinoma cells (purchased from the National Infrastructure of Cell Line Resource, Beijing, China) were grown in RPMI 1640 medium (Gibco, 31800-022) supplemented with 10% (*v*/*v*) fetal bovine serum (FBS), 100 U/mL penicillin, 100 mg/mL streptomycin, 10 mM HEPES, 1 mM sodium pyruvate, and 50 μM β-mercaptoethanol in a humidified atmosphere containing 95% air and 5% CO_2_. Culture medium was replaced every 2–3 days. After reaching near confluence, cells were treated with 0.25% trypsin and 0.02% EDTA for 1 min and replated into 55 cm^2^ flasks. The cytotoxicity of tigerinin-1R analogs was determined by MTT assay. Cells were transferred into 96-well plates (5 × 10^3^ cells/well) and incubated with serially diluted concentrations of different peptides (10^−8^, 10^−6^, 10^−4^ M) for 24 h at 37 °C. Meanwhile, cells without the addition of the peptides were set as the negative control. At the end of incubation, 20 μL of MTT solution (5 mg/mL in PBS) was added to each well and further incubated at 37 °C for 4 h. Cell viability was determined by dissolving the crystallized MTT with dimethyl sulfoxide (150 μL/well). The absorbance at 492 nm was measured using a microplate reader (GF-M3000; Gaomi Caihong Analytical Instruments Co., Ltd., Gaomi, China). The MTT assays were repeated in triplicate.

### 4.5. Determination of Insulin-Releasing Activity In Vitro

INS-1 cells were harvested with trypsin/EDTA, seeded into 6-well plates (3–5 × 10^5^ cells/well), and allowed to attach overnight. Prior to the test, cells were preincubated in 1 mL Krebs-Ringer bicarbonate (KRB) buffer without glucose (115 mMNaCl, 4.7 mMKCl, 1.28 mM CaCl_2_,1.2 mM KH_2_PO_4_, 1.2 mM MgSO_4_, 10 mM NaHCO_3_, and 1 g/L BSA, pH 7.4) at 37 °C for 45 min. Cells were then incubated in the absence or presence of peptides for 1 h, using the same buffer with 2.8 mM glucose [35]. The insulin supernatants were collected, and subsequently analyzed using ultrasensitive rat insulin ELISA kits (Mercodia, Sweden). The assay was repeated in triplicate.

### 4.6. Lactate Dehydrogenase Leakage Assay

INS-1 cells (5 × 10^3^) were seeded in 96-well plates for 24 h and then incubated with 100 μL of KRB buffer supplemented with 2.8 mM glucose containing10^−5^ M peptide. Untreated cells were used as the negative control (0% leakage). Cells incubated with 1% Triton X-100 served as the positive control (100% leakage). Data were measured at 450 nm. LDH leakage rate (% of positive control) was respectively calculated. 

### 4.7. Measurement of [Ca^2+^]

INS-1 cells (2 × 10^5^) were cultured in glass bottom dishes (NEST, Wuxi, China). Following overnight culture, the cells were washed with KRB buffer three times, and then incubated with 5 μM fluorescent Ca^2+^ probe Fluo-3 AM solution (Solarbio, Beijng, China). After loading for 30 min and washing three times with KRB buffer, cells were incubated for 15 min in the absence of Fluo-3 AM. KRB buffer supplemented with 2.8 mM glucose containing 10^−5^ M peptide was then added. Images of cells were obtained by laser scanning confocal microscope (LSM710, Carl Zeiss, Oberkochen, Germany). The fluorescence was excited at 480 nm at 4 s intervals, and detected at 515 nm. Incubations in the presence of 2.8 mM glucose alone (negative control) or extra 30 mM KCl (positive control) were also carried out individually.

### 4.8. In Vivo Studies

Kunming male mice were housed individually in an air-conditioned room (22 ± 2 °C) with a 12 h light/12 h dark cycle, and were handled in accordance with the Guide for Care and Use of Laboratory Animals in China and approved by the animal ethics committee of Jilin University (Approval No. JLUSWLL003, Jilin, China; dated on 10 May 2017). Drinking water and standard laboratory chow were freely available. No adverse effects were observed following administration of the peptides. Age-matched groups (*n* = 6) of overnight-fasted mice received an intraperitoneal injection of either glucose (18 mmol/kg body weight), tigerinin-1R (75 nmol/kg body weight), or [C11K]tigerinin-1R (75 nmol/kg body weight) individually as control groups, and glucose in combination with tigerinin-1R or glucose in combination with [C11K]tigerinin-1R as experimental groups, respectively. All solutions were administered in 0.9% NaCl (5 mL/kg body weight) [41]. Blood samples were collected from the cut tip of the tail vein of conscious mice, before injection and at 15, 30, 60, and 120 min immediately after injection, and glucose was measured using a Sannuo stable blood glucose meter (Changsha, China) using corresponding test strips.

### 4.9. Peptide Degradation Assays

Peptides dissolved in PBS to a final concentration of 200 μM were incubated with 10% fetal bovine serum at 37 °C. At different time points (0, 60, and 120 min), 20 μL peptide samples were taken and diluted with an equal volume of 0.1% aqueous trifluoroacetic acid (TFA). The samples were then analyzed by RP-HPLC. The experiment was performed on a Shimadzu HPLC system with a Zorbax 300 SB-C8 column (150 × 4.6 mm I.D.; 5 µm particle size, 300 Å pore size; Agilent Technologies, Palo Alto, CA, USA) using a linear AB gradient (1% acetonitrile/min) and a flow rate of 1 mL/min, where solvent A was 0.1% aqueous TFA, pH 2, and solvent B was 0.1% TFA in acetonitrile at 25 °C.

### 4.10. Apoptosis Induction and Cell-Viability Assay

Solutions of free fatty acids (FFAs) were prepared [8]. Briefly, 100 mM palmitic acid (PA) stocks (Sigma, St. Louis, MO, USA) were prepared in 0.1 M NaOH at 70 °C. Ten percent (*w*/*v*) FFA-free BSA (Roche, Mannheim, Germany) solution was prepared in PBS buffer. A 5 mM FFA/10% BSA solution was prepared by mixing 50 μL FFA to 950 μL 10% BSA in a 60 °C water bath. The above solution was then cooled to room temperature, diluted in RPMI 1640 to 0.5 mM and filtered. Cells were added into 96-well plates (5 × 10^3^ cells/well). Complete medium was withdrawn after overnight incubation at 37 °C. One hundred microliters of different peptides corresponding to a serial dilution series (concentrations 10^−9^, 10^−7^, 10^−5^ M) diluted with medium containing 0.5 mM PA, was added for another 24 h. Meanwhile, incubation in fresh medium, with or without PA, were set as positive and negative controls, respectively. At the end of incubation, 20 μL of MTT solution (5 mg/mL in PBS) was added to each well and further incubated at 37 °C for 4 h. Cell viability was determined by dissolving the crystallized MTT with dimethyl sulfoxide (150 μL/well).

### 4.11. Hoechst 33258 Staining

Hoechst 33258 staining was also performed to evaluate the protective effects of the peptides. INS-1 cells were seeded in 12-well plates (1 × 10^5^ cells/well) and allowed to attach overnight. After incubation with different peptides (dissolved in RPMI 1640 contained 0.5 mM PA) for 24 h, photos were taken by fluorescence microscopy (LX71, Olympus, Kawasaki, Japan). Medium without peptides in the presence/absence of PA were used as positive or negative controls, respectively.

## 5. Conclusions

In conclusion, the role of disulfide bond in the activity and stability of tigerinin-1R was explored in this study. Disulfide bond is important but not irreplaceable to in vitro activities, and crucial due to the contribution of stability to in vivo activities, especially with regard to glucose tolerance activity. Tigerin-1R peptide analogs stimulated insulin release through a mechanism that involves the mobilization of intracellular calcium in INS-1 cells, confirming the universality of the mechanism of insulinotropic activity. It is noteworthy that tigerinin-1R peptides can protect pancreatic β-cells from damage caused by oxidative stress. Thus, tigerinin-1R may represent a promising candidate as an anti-diabetic therapeutic agent in clinical practices.

## Figures and Tables

**Table 1:** Sequence and biophysical data of peptides used in the study.

No.	Peptide	Amino Acid Sequence ^a^	Mw	*t*_R_ (min) ^b^
1	tigerinin-1R	RVCSAIPLPICH-amide (C–C)	1035.65	19.465
2	tigerinin-1R-L4	RVCS*LL*PLP*L*CH-amide (C–C)	1347.73	23.608
3	tigerinin-cyclic	CSAIPLPIC-amide (C–C)	955.23	19.647
4	tigerinin-linear	Ac-RV*A*SAIPLPI*A*H-amide	1285.56	11.590
5	[C3K]tigerinin-1R	Ac-RV*K*SAIPLPICH-amide	1374.72	10.818
6	[C11K]tigerinin-1R	Ac-RVCSAIPLPI*K*H-amide	1374.72	8.835

^a^ Peptide sequences are shown by using the one-letter code for amino acid residues; Ac, N-acetyl; amide, C-terminal amide; C–C in parentheses means that the two Cys residues in the sequences form intermolecular disulfide bond; the bold and italic letters denote the substituting amino acids. ^b^ *t*_R_ (min) denotes the retention time of the peptides at 25 °C during reversed-phase HPLC.
